# Gene expression and glycan profiling of CD4+T cells in HAM/TSP

**DOI:** 10.1186/1742-4690-8-S1-A197

**Published:** 2011-06-06

**Authors:** Daisuke Kodama, Ryuji Kubota, Shuji Izumo

**Affiliations:** 1 Molecular Pathology, Center for Chronic Viral Diseases, Kagoshima University Graduate School of Medical and Dental Sciences, Kagoshima-Shi, 890-8544, Japan

## 

Gene expression profile (transcriptome) and glycan profile (glycome) of HTLV-1-infected CD4+T cells may reflect the pathologic cellular mechanism and intercellular recognition, respectively in HAM/TSP though these profiles are still not obtained. To identify responsible cellular genes and relevant glycans of HAM/TSP, we performed experiments with microarray on RNAs and and lectin array on proteins extracted from CD4+Tcells from each four subjects of three goups including HAM/TSP, asymptomatic carriers (AC), and HTLV-1 negative controls (NC). In transcriptome analysis, transcripts of 177 genes were found up-regulated only in HAM/TSP, and those may possibly be causative or resultant genes. In glycome analysis with lectin array carrying 45 species of lectins, standardized signals of UDA (Urtica dioica agglutinin, stinging nettle) and STL (Solanum tuberosum lectin, potato) which recognize N-glycan (GlcNAc)n were significantly high in samples from HTLV-1 infected groups (HAM/TSP and AC) compared with NC. Interestingly, UDA has been recently reported to inhibit cell-to-cell transmission of HTLV-1 in vitro. These genes and glycans may play roles in the pathogenesis of HAM/TSP.

